# Occurrence of *Salmonella* spp.: a comparison between indoor and outdoor housing of broilers and laying hens

**DOI:** 10.1186/s13028-017-0281-4

**Published:** 2017-02-21

**Authors:** Martin Wierup, Helene Wahlström, Elina Lahti, Helena Eriksson, Désirée S. Jansson, Åsa Odelros, Linda Ernholm

**Affiliations:** 1 Department of Biomedical Sciences and Veterinary Public Health, Swedish University of Agricultural Sciences, P.O. Box 7028, SE-75007 Uppsala, Sweden; 2 Department of Disease Control and Epidemiology, National Veterinary Institute, SVA, SE-751 89 Uppsala, Sweden; 3 Åsa Odelros AB, Österåkersvägen 21, SE-81040 Hedesunda, Sweden; 4 Department of Animal Health and Antimicrobial Strategies, National Veterinary Institute, SVA, Österåkersvägen 21, SE-81040 Hedesunda, Sweden

**Keywords:** *Salmonella*, Broiler, Laying hen, Outdoor poultry production, Indoor poultry production, Environmental exposure, Wildlife, Free-range

## Abstract

**Background:**

Outdoor production of poultry is rapidly increasing, which could be associated with increased risks for exposure to different environmental sources of *Salmonella*. We report a comparison on the occurrence of *Salmonella* during 2007–2015 in broilers and laying hens in outdoor and indoor production subjected to the same requirements for the prevention and control of *Salmonella* as applied in Sweden.

**Results:**

Our results give no indication that, during the period studied, the exposure to *Salmonella* in outdoor poultry production was higher than in the indoor production. The annual incidence of *Salmonella* infected flocks in outdoor production remained at a very low and at a similar level as for indoor production. For laying hens the annual proportion of birds in test positive flocks ranged from 0 to 1.3% for indoor production from 0 to 2.0% for outdoor production. For broilers the proportion of *Salmonella* infected flocks (2013–2015) was 0.16% for indoor, and 0% in outdoor production. The difference was not statistically significant and was further reduced when flocks infected due to vertical transmission or from a hatchery source were excluded. It should, however, be considered that the number of outdoor flocks included in this evaluation is very small and continuous evaluation is needed.

**Conclusions:**

New animal production systems, including those driven by consumer and welfare demands, may be associated with a higher risk for the exposure of potential pathogens to food animals and possibly also subsequent outbreaks of food borne infections. In this study no increase in the risk for exposure of flocks to *Salmonella* in outdoor poultry production was found. The situation may well change and the possibility of *Salmonella* contamination in outdoor poultry production requires continuous attention.

## Background

*Salmonella* is a major food borne pathogen which globally is estimated to cause 93 million enteric infections and 155,000 diarrheal deaths each year [1]. Poultry products are a significant source which initially was considered to be a consequence of the global introduction of industrialized production of broiler chickens around some 50 years ago [2]. In the late 1980s, the emerging and pandemic spread of *Salmonella* Enteritidis primarily via table eggs also focused attention on the presence of *Salmonella* in the laying hen industry [3]. This pandemic reached alarming proportions and, e.g. in Germany, it was estimated that two million human food-borne *Salmonella* infections occurred annually, of which the majority were caused by serovar Enteritidis [4]. Efforts were therefore made to prevent the spread of *Salmonell*a in particular from the poultry industry, and in the EU a significant decreasing trend of human cases of salmonellosis has been observed mainly attributed to successful implementation of national *Salmonella* control programs at the preharvest level in poultry populations [5]. Nevertheless, poultry meat remained the food product from which *Salmonella* was most frequently detected and eggs are still the most important source of reported outbreaks of food-borne salmonellosis [5].

Long term experience from Sweden, Finland and Norway has shown that exposure of poultry to *Salmonella* can largely be prevented in indoor production of broiler chickens and in laying hens [6, 7]. In these countries, the prevalence of *Salmonella* of any serovar is extremely low [5]. However, outdoor production of poultry is rapidly increasing in Sweden, which could be associated with increased risks for exposure to different environmental sources of *Salmonell*a, including wildlife [8–10]. It could further be assumed that cleaning and disinfection applied between flocks in indoor production and in particular when outbreaks of *Salmonella* infections have occurred would be less efficient in minimizing the risk for residual *Salmonella* contamination in outdoor conditions.

The objective of this study was to analyze the risk for exposure of *Salmonella* in outdoor poultry production compared to indoor production. The results should also indicate if methods successfully applied for the prevention and control of *Salmonella* in indoor poultry production are equally useful under outdoor production conditions.

## Methods

### Control measures for *Salmonella*

This study is based on results from the official Swedish control of *Salmonella* and associated testing procedures, which are similar for indoor and outdoor production. A voluntary preventive *Salmonella* control program for poultry has been in place since 1970. In 1984 pre-slaughter testing of broiler flocks became mandatory. In response to the pandemic spread of *Salmonella* Enteritidis during the late 1980s a voluntary control program based on pre-slaughter sampling was initiated for laying hens in 1990. In 1994 sampling of laying hen flocks became mandatory not only before slaughter but also during the production period [11]. The programme was further intensified in 1995 when Sweden joined the European Union. In case of findings of *Salmonella*, regardless of serovar, the affected flocks (epidemiological unit) are euthanized, followed by cleaning and disinfection of the poultry holding premises and repeated *Salmonella* sampling of the environment, which has to be negative before restocking.

Currently, sampling for *Salmonella* in Swedish poultry flocks is performed as described in the EU harmonized regulations (breeders of *Gallus gallus* EU 200/2010, laying hens of *Gallus gallus* 517/2011, broilers EU 200/2012) with some exceptions. All poultry flocks delivering birds to an abattoir, irrespectively of the flock size, are tested 1–2 weeks before slaughter. In addition, all laying hen flocks are tested once during the rearing period and every 15th week during the production period as well as before slaughter, i.e. usually four to five times during the production period. Samples are taken from all sections of the poultry house. For non-cage systems, the test material consists of two pairs of sock samples in production flocks, five pairs of sock samples in breeding flocks, while for flocks housed in enriched cages faecal samples (2 × 75 g) are collected [12]. The results must be available before slaughter and only test negative flocks are allowed to deliver table eggs to egg packing plants and birds for slaughter. Before the above described controls were implemented for broilers and laying hens, specific requirements for control of *Salmonella* were in place for the breeding flocks as well as for the poultry feed [13].

Only accredited laboratories are allowed to perform the analyses. All samples from animals including poultry are analyzed using the MSRV (EN-ISO 6579:2002/A1: 2007: Amendment 1: Annex D) method. Putative isolates of Salmonella are sent for confirmation, serotyping, antimicrobial susceptibility testing and other typing to the National Veterinary Institute (SVA). The results from the control of Salmonella are reported and are presented annually in National Zoonosis Reports and the EU harmonized data is also included in the EFSA/ECDC´s zoonosis reports.

The results of the monitoring of *Salmonella* in laying hens and broilers are based on the testing of flocks i.e. the epidemiological unit of birds defined for each production holding.

### Population and study period

The study was limited to chickens (*Gallus gallus* dom.) i.e. laying hens producing table eggs (including the rearing period up to 16 weeks of age) and broiler chickens. In this study a flock was considered to belong to an outdoor system if the birds during any time period had had access to the outdoor environment. Apart from that separation, all indoor laying hens were considered as equal although different housing systems exist as recently have been described [14]. Breeder flocks (grandparent and parent flocks) were not included since outdoor production is not allowed in this population. The study covered 9 years (2007–2015).

### Data sources

Data on the numbers of slaughtered broilers were retrieved from official statistics of the Swedish Board of Agriculture. Data on the laying hen population in terms of housing capacity i.e. maximum total number of laying hens at one time in all Swedish laying hen houses were provided by The Swedish Egg Association. The total number of slaughtered broiler flocks (>350 birds) representing the number of *Salmone*lla—tested flocks, was available for 2013–2015 from the Swedish Poultry Meat Association. Information on whether individual laying hen or broiler flocks were reared indoors or outdoors was obtained through the Swedish Egg Association, The Swedish Poultry Meat Association; and from the organization for organic farming KRAV. Data on *Salmonella*—infected flocks of laying hens and broilers was obtained from the official statistics. Further information on the number of birds in each infected flock and if the flock was housed indoors or outdoors were obtained from reports of outbreak investigations by the Swedish Board of Agriculture and the National Veterinary Institute.

### Statistical methods

#### Laying hens

To obtain an estimate of exposure to *Salmonella* that was comparable between indoor and outdoor production, the number of birds in infected flocks was divided by the respective total housing capacity for indoor and outdoor production. These calculations were made on an annual basis and also for the whole period. For the latter, the nominator was the number of birds in test positive flocks during 2008–2015 (for indoor and outdoor production, respectively) and the denominator was the sum of the total of housing capacity each year for indoor and outdoor production. For year 2010, where data on housing capacity was missing, an average of 2009 and 2011 was used.

#### Broilers

An estimate of the exposure to *Salmonella* that was comparable between the in-and outdoor production was obtained by dividing the number of birds in test-positive flocks, i.e. the unit of concern where sampling is done, by the total number of slaughtered birds raised in the production specific systems. The latter calculations were made annually for years 2007–2015 and for the whole period. A second estimate of exposure was obtained by calculating the proportion of test-positive flocks in indoor and outdoor production. As data on flock level was only available from 2013, calculations were made for the years 2013–2015 and for the whole period. As all flocks were sampled, there is no random variation for the flock prevalence estimates for each of years 2013–2015. However, to estimate the uncertainty, if these flocks are considered to be samples from the population of outdoor broiler flocks, the exact 95% confidence intervals were calculated for the proportion of test positive flocks using binom test, stats package, R version 3.1.1.

## Results

### Laying hen production

**Fig. 1:**
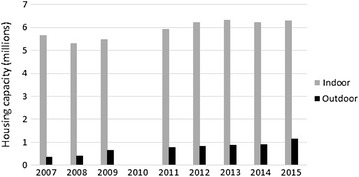
Total number of laying hens in Sweden by indoor and outdoor production

Between 2007 and 2015, the total number of laying hens (Table 1; Fig. 1) increased from 6.0 million birds to 7.4 million birds (24%). The proportion of outdoor production (out of the total production) increased from 5.9% in 2007 to 15.5% in 2015. Outdoor production increased both in absolute numbers as a proportion of the total housing capacity.

**Table 1:** Flocks of laying hens tested positive for *Salmonella* in indoor and outdoor production in Sweden

Year	Housing capacity^a^		No. test-positive flocks		No. birds in test-positive flocks		No. birds in test-positive flocks/total housing capacity (%)	
	Indoor	Outdoor	Indoor	Outdoor	Indoor	Outdoor	Indoor	Outdoor
2007	5,649,000	351,000	1	2	Nd^b^	Nd^b^	Nd^b^	Nd^b^
2008	5,285,639	413,305	5	0	66,900	0	1.3	0.0
2009	5,457,711	646,930	1	2	20,000	2820	0.4	0.4
2010	5,690,353	708,435	2	0	23,600	0	0.4	0.0
2011	5,922,995	769,939	0	0	0	0	0.0	0.0
2012	6,222,224	816,864	2	0	17,600	0	0.3	0.0
2013	6,312,001	875,437	7	0	69,800	0	1.1	0.0
2014	6,217,685	896,371	1	1	6600	18,000	0.1	2.0
215	6,289,060	1,153,615	2	0	39,399	0	0.6	0.0
Total^c^ 2008–2015	47,397,668	6,280,895	20	3	243,899	20,820	0.5	0.3

^a^Maximum total number of laying hens at one time in laying hen houses

^b^Not available

^c^Year 2007 not included since data on number of birds in test-positive flocks was missing

The results of the control of *Salmonella* in laying hens are presented in Table 1. The numbers of infected flocks ranged from zero to seven flocks annually. In total, 26 flocks were found to be *Salmonella*-infected during the 9-year period (2007–2015) and five of these flocks (19%) were from outdoor production. No rearing flocks were found to be *Salmonella* infected (data not shown). The proportion of birds in test-positive flocks/total housing capacity during the period studied ranged from 0 to 1.3% for indoor production and from 0 to 2.0% for outdoor production on an annual basis. For the whole period the corresponding data was 0.5% for indoor production and 0.3% for outdoor production (Table 1).

### Broiler production

**Fig. 2:**
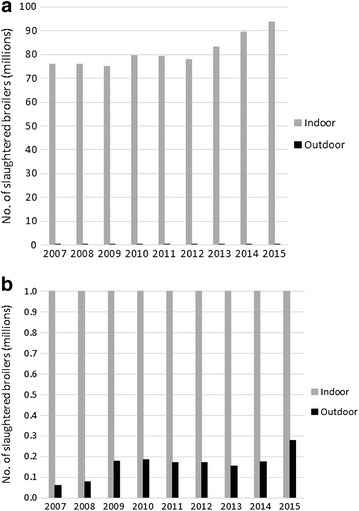
Total number of broilers slaughtered from indoor and outdoor production in Sweden (**a**) and the outdoor production further visualized using truncated *Y*-*axis* (**b**)

The annual production of broiler chickens (no of slaughtered birds) was 94 million in 2015, which represents an increase by 24% since 2007 (Table 2; Fig. 2a). The majority of birds were raised indoors (99.7% in 2015) but the outdoor production increased from 0.06 million 2007 to 0.28 million in 2015 (Fig. 2b).

**Table 2:** Flocks of broilers tested positive for *Salmonella in* indoor and outdoor production in Sweden

Year	No. slaughtered birds		No. slaughtered flocks		No. test-positive flocks		No. birds in test-positive flocks		No. birds in test-positive flocks/no. slaughtered birds (%)		Proportion test-positive flocks (%)	
	Indoor	Outdoor	Indoor	Outdoor	Indoor	Outdoor	Indoor	Outdoor	Indoor	Outdoor	Indoor	Outdoor
2007	75,987,684	62,040	Nd^a^	Nd^a^	10^b^	1	186,500	1400	0.25	2.26	Nd^a^	Nd^a^
2008	76,029,408	79,055	Nd^a^	Nd^a^	8	0	206,700	0	0.27	0.00	Nd^a^	Nd^a^
2009	74,836,094	179,050	Nd^a^	Nd^a^	1	3	35,000	4070	0.05	2.27	Nd^a^	Nd^a^
2010	79,413,074	185,020	Nd^a^	Nd^a^	17^c^	0	320,555	0	0.40	0.00	Nd^a^	Nd^a^
2011	79,193,063	170,030	Nd^a^	Nd^a^	3	1	47,000	400	0.06	0.24	Nd^a^	Nd^a^
2012	77,903,897	170,045	Nd^a^	Nd^a^	1	0	31,000	0	0.04	0.00	Nd^a^	Nd^a^
2013	83,110,440	155,000	3233	43	1	0	61,800	0	0.07	0.00	0.03	0.0
2014	89,504,467	176,500	3232	44	2	0	55,000	0	0.06	0.00	0.06	0.0
2015	93,820,000	280,000	3329	61	13^d^	0	405,574	0	0.43	0.00	0.39	0.0
Total	729,798,117	1,456,740	9794	148	56	5	1,349,129	5870	0.18	0.40	0.16	0.0

^a^Not available

^b^Three of the flocks originated from a *Salmonella*—infected breeding flock

^c^15 of the flocks originated from a *Salmonella*—contaminated hatchery

^d^Six of the flocks originated from a *Salmonella*—infected breeding flock

The results of the control of *Salmonella* are presented in Table 2. The numbers of test-positive flocks were low and ranged from 1 to 17 flocks annually. In total, 61 flocks were found to be infected with *Salmonella* during the 9-year period (2007–2015), of which five flocks (8.2%) were raised outdoors. In 2007 and 2015, three and six of the infected broiler flocks, respectively, could be epidemiologically linked to infected breeding flocks and during 2010, 15 of the infected flocks originated from the same breeding company, hatched very closely in time. For the years when data was available (2013–2015) the proportion of infected broiler flocks was higher in indoor production (0.16%, 95% CI 0.09–0.2%) than in outdoor production (0%, 95% CI 0–2%), but this was not significantly different (Table 2). When excluding the six flocks which during 2015 were linked to infected breeding flocks, the proportion of *Salmonella* test-positive broiler flocks in indoor production during 2013–2015 decreased from 0.16 to 0.10%.

The proportion of the total number of animals in *Salmonella* test-positive flocks out of the total of animals slaughtered, divided by indoor and outdoor production was calculated for the years 2007–2015 (Table 2). This proportion was (0.18%) in flocks raised indoors compared to (0.4%) in flocks with access to outdoor conditions.

### Isolated *Salmonella* serovars

The isolated serovars of *Salmonella* are listed in Table 3. Only one serovar or subspecies was isolated from each of the 87 infected flocks. In both indoor and outdoor production *Salmonella* Typhimurium was the most common serovar and accounted for 67 and 60% of all the isolates respectively. *Salmonella* Typhimurium was also the only serovar involved in the dissemination of *Salmonella* from parent flocks or hatchery to 24 broiler flocks. All six flocks infected with serovar Agona were recurrently infected broiler flocks housed on the same farm. Infections with serovar Livingstone all involved laying hen flocks at four different farms and in two of these the serovar reoccurred. These reoccurring infections all concerned indoor production.

**Table 3:** *Salmonella* isolated from laying hens and broilers in indoor and outdoor production in Sweden

Serovar or subspecies of *Salmonella*	No. isolates/flock 2007–2015^a^	
	Indoor production	Outdoor production
Agona	6^b^	0
Be	0	1
diarizonae (IIIb) serovar O38:r:z	1	1
Epinay	2	0
Goldcoast	0	2
Livingstone	9	0
Mbandaka	3	0
Meleagridis	2	0
Reading	2	0
Typhimurium	52	6
Total	77	10

^a^Only one serovar was isolated from each of the 87 flocks tested positive for *Salmonella*

^b^All six Agona isolates originated from the same farm

## Discussion

In this study, the occurrence of *Salmonella* for indoor and outdoor housing of laying hens and broilers was analyzed. The method applied was to compare the occurrence of *Salmonella* test-positive flocks and also the proportion of birds in test-positive flocks (broilers and laying hens) in outdoor and indoor production subjected to similar requirements for the prevention and control of *Salmonella*.

Our results gave no indication that, during the period studied, the exposure to *Salmonella* in outdoor poultry production was higher than in indoor production. In both the outdoor laying hen and broiler production the annual incidence of *Salmonella*-infected flocks remained at a very low level and at a similar level as for indoor production. For laying hens, data at flock level was not available but the proportion of birds in test positive flocks was not higher in outdoor production. For broilers, where data on flock level was available, there was no significant difference between indoor and outdoor production. The proportion of the total number of birds in Salmonella test-positive flocks at slaughter were low, although slightly higher in outdoor production. The overall conclusion was that there is no indication that the risk of Salmonella is much higher in outdoor production in Sweden. It should however be considered that the number of outdoor flocks included in this evaluation is very small and that the risk may vary over time. It is therefore recommended that the situation should be continuously evaluated.

It should be noted that the incidence of *Salmonella* -infected broiler flocks was relatively high for Swedish circumstances and with a substantial variation between years. This variation can largely be explained by vertical transmission from *Salmonella* infected parent flocks in 2007 and 2015 and a *Salmonella*—contaminated hatchery in 2010. Vertical spread of *Salmonella* from the parent stock, which previously has been an extremely rare event in Swedish commercial poultry production, does not reflect the risk for *Salmonella* contamination related to indoor or outdoor production at the production level and this was therefore also considered in the assessment. However, the broilers with outdoor access predominantly originate from the same breeding companies as the indoor broilers. During the period of the study the same genotypes were used for both indoor and outdoor production for both broiler and laying hens respectively. The fact that no outdoor flock was infected by these issues may reflect the small proportion of outdoor production.

The risk for *Salmonella* contamination in outdoor production merits continuous attention, although the results from Sweden so far do not suggest that the current methods for prevention and control of *Salmonella* have to be modified for outdoor production. It should primarily be noted that the period studied represents the early stage of a current trend towards commercial production of poultry outdoors as recently described [14]. In the future, the risk for exposure to *Salmonella* in outdoor production may change. Secondly, an increased risk can follow on infected farms if residual *Salmonella* contamination cannot be eliminated by the sanitary methods that are successfully applied in indoor production. So far, our corresponding knowledge for outdoor production is limited and requires further attention, in particular decontamination of outdoor runs and natural ground surfaces. Methods for preventing contact and contamination from wildlife, mainly passerine birds, are also of importance. Apart from passerine birds and in certain areas hedgehogs, *Salmonella* in wild-life in Sweden is very rare [12, 15] but the situation can be considerably different in other countries [10]. The location of outdoor poultry production in areas with a high density of farm animal populations, particularly in countries with less stringent control of *Salmonella* in pigs and cattle can be an additional factor that may significantly influence the risk for *Salmonell*a -infection in outdoor production. In order to minimize the risk for exposure of *Salmonella* to the outdoor production, basic knowledge on biosecurity, including prevention and control of *Salmonella* is, essential for new producers and farm staff.

The isolated serovars of *Salmonella* included those generally isolated from poultry and animal feed ingredients [12]. Independent of housing conditions, Typhimurium was the most commonly isolated serovar. Further subtyping by e.g. multi-locus variable number tandem repeat analysis (MLVA) can reveal additional epidemiological information on the source of infection [10]. Recurrent infections may thus be linked to certain strains, as was observed in this study for Agona where one farm with indoor broilers had infected flocks recurring six times over a period of two and a half years. Recurrent infections could also be seen for other serovars in our study, but not to the same extent. However, in our study only one outdoor holding has shown a repeated *Salmonella*—infection during the studied period. The test-positive flocks at that holding were of different categories, one in laying hens and one in broilers and were spaced 2 years apart. Both flocks were infected with *S. Typhimurium* but of different phage types, RDNC for broilers and U277 for laying hens, and are therefore more likely to result from separate introductions of infection.

For various reasons it is difficult to compare the result of our studies with those of most others found in the literature. Some previous studies have described the impact of different laying hen housing conditions on the prevalence of *Salmonella*, in particular prior to the 2012 ban in the European Union of housing of laying hens in conventional battery cages. However, due to many different risk factors involved including e.g. flock size, methods for cleaning and disinfection between batches and methods of sampling, it is difficult to draw detailed conclusions concerning risks for *Salmonella* infection in poultry in outdoor conditions [16–18]. A major limiting factor for an evaluation and comparison with the results of our study is that the referred studies generally were performed under what could be called high *Salmonella* prevalence conditions in flocks where active specific control of *Salmonella* was limited or absent as previously observed in studies on *Salmonella* in the pig production [19]. In the absence of such control, the prevalence of *Salmonella* is generally higher, which was demonstrated in a comprehensive EU baseline study based on a harmonized sampling from 5310 poultry holdings in 24 Member States. In the EU study, *Salmonella* was detected in 30.8% of the laying hen holdings [6]. That study also found that in the Member States, the observed flock prevalence of *Salmonella* ranged from 0 to 79.5%. The lowest prevalence figures were observed in countries including Sweden, with a long history of active control of *Salmonella*. However, in an individual country production system with special control measures for *Salmonella* can be applied. Recent data from France reports a decreasing trend of *Salmonella* contamination in outdoor production in the traditional free-range “Label Rouge” broiler production [20]. The prevalence of *Salmonella*—contaminated carcasses decreased from 16 to <2% during 1994–2014, and a prevalence of 1.47–2.65% *Salmonella*—infected flocks was achieved during 2010–2014, although this is higher than in this study.

Interestingly, in the present study, only one serovar of *Salmonella* was isolated from each of the 87 flocks found infected. This fact most likely also reflects that this study was performed in a low prevalence country were the serovar diversity of environmental *Salmonella* contamination, in particular around animal farms, can be expected to be lower than in countries where the implementation of preventive measures and control of *Salmonella* has been more limited. It is here also interesting to compare with *Campylobacter*, another highly important zoonotic poultry associated pathogen. In Sweden a significant difference have been found in the prevalence of *Campylobacter* in caecum of conventionally indoor produced broilers (13%) and broilers produced in organic and other small scale production systems with outdoor access (60%) [21]. Because the epidemiology of *Campylobacter* is different from *Salmonella* and only partly understood, there in contrast to *Salmonella* are currently no identified measures for the reliable control of this organism in free ranged poultry [22].

## Conclusions

In summary, new animal production systems, including those driven by consumer and welfare demands may potentially be associated with a higher risk for the exposure of potential pathogens to food animals and possibly also subsequent outbreaks of food-borne infections. In order to prevent such scenarios, new production systems require special attention and monitoring so necessary actions can be taken should such risks incursions occur. In this study, no increase in the risk for exposure of *Salmonella* in outdoor poultry production was found so far, despite the current trend towards such production conditions. However, this situation may well change and opportunity for *Salmonella* -contamination in the outdoor poultry production requires continuous attention.
